# Usutu Virus: An Emerging Flavivirus in Europe

**DOI:** 10.3390/v7010219

**Published:** 2015-01-19

**Authors:** Usama Ashraf, Jing Ye, Xindi Ruan, Shengfeng Wan, Bibo Zhu, Shengbo Cao

**Affiliations:** 1 State Key Laboratory of Agricultural Microbiology, Huazhong Agricultural University, Wuhan, Hubei 430070, China; E-Mails: usamaashraf1523@gmail.com (U.A.); yeah_zaizai@hotmail.com (J.Y.); 258390599@qq.com (X.R.); wansf126@126.com (S.W.); zhubibohzau@126.com (B.Z.); 2 Laboratory of Animal Virology, College of Veterinary Medicine, Huazhong Agricultural University, Wuhan, Hubei 430070, China; 3 Laboratory of development of Veterinary Diagnostic Products, Ministry of Agriculture, College of Veterinary Medicine, Huazhong Agricultural University, Wuhan, Hubei 430070, China

**Keywords:** Usutu virus, SouthAfrica-1959, Austria, *Culex pipiens*, *Turdus merula*

## Abstract

Usutu virus (USUV) is an African mosquito-borne flavivirus belonging to the Japanese encephalitis virus serocomplex. USUV is closely related to Murray Valley encephalitis virus, Japanese encephalitis virus, and West Nile virus. USUV was discovered in South Africa in 1959. In Europe, the first true demonstration of circulation of USUV was reported in Austria in 2001 with a significant die-off of Eurasian blackbirds. In the subsequent years, USUV expanded to neighboring countries, including Italy, Germany, Spain, Hungary, Switzerland, Poland, England, Czech Republic, Greece, and Belgium, where it caused unusual mortality in birds. In 2009, the first two human cases of USUV infection in Europe have been reported in Italy, causing meningoencephalitis in immunocompromised patients. This review describes USUV in terms of its life cycle, USUV surveillance from Africa to Europe, human cases, its cellular tropism and pathogenesis, its genetic relationship with other flaviviruses, genetic diversity among USUV strains, its diagnosis, and a discussion of the potential future threat to Asian countries.

## 1. Introduction

The genus *Flavivirus* of family *Flaviviridae* is composed of more than 70 viruses. Among them, Japanese encephalitis virus (JEV), West Nile virus (WNV), Murray Valley encephalitis virus (MVEV), dengue virus (DENV), St. Louis encephalitis virus, and yellow fever virus are important threats to human health [1,2,3]. Usutu virus (USUV) is a mosquito-borne flavivirus belonging to the JEV serocomplex [2,4] and thus is closely related to JEV, MVEV, and WNV [4]. In 1959, USUV was isolated from *Culex neavei* mosquitoes in South Africa, and this strain, SouthAfrica-1959, is now considered as the reference strain [5]. Later, USUV was found to be associated with fever and rash in an African man [6]. In Europe, the first emergence of USUV was reported in Austria in 2001 [7]; however, retrospective analysis of archived tissue samples from bird deaths in the Tuscany region of Italy in 1996 [8] proved a much earlier introduction of USUV into Europe than previously assumed [9]. In the subsequent years, USUV was found to circulate in several other European countries by mosquitoes displacement or infected birds [10,11]. In comparison to the human USUV case in Africa, the human cases in Europe were more serious with typical flavivirus-related neuroinvasiveness and neurovirulence [12,13].

This review focuses on aspects of USUV related to its emergence from Africa and spread to Europe, as well as genetic diversity among different USUV strains.

## 2. Life Cycle of USUV

The life cycle of USUV is quite similar to that of other members of the JEV serocomplex. Its natural life cycle involves mosquito-bird-mosquito cycles, in which mosquitoes act as vectors and birds as amplifying hosts. Many studies have demonstrated that multiple mosquito and avian species are involved in perpetuating the USUV life cycle [10,11]. Mosquitoes facilitate viral transmission to humans, horses, and rodents, which then act as incidental hosts [6,12,13,14]. Recently, USUV has also been isolated from bats in Germany [15]. The detection of USUV in bats raised questions for future research, including the potential role of bats as reservoirs in Africa and transmission by mosquito vectors.

USUV has been isolated from numerous mosquito species that include *Culex pipiens* [16,17,18,19,20,21], *Cx. neavei* [5], *Culex perexiguus* [10], *Aedes albopictus* [16], *Aedes caspius*, *Anopheles maculipennis* [19], *Culex perfuscus*, *Coquillettidia aurites*, and *Mansonia Africana* [22,23]. Of these, *Cx. pipiens* is considered to be the most common vector [16,17,18,19,20,21]. In addition, *Cx. neavei* is the only mosquito species whose vector competence for USUV is known [24]; therefore, vector competence studies involving other mosquito species should be done to confirm their vector status.

Among avian species, Eurasian blackbirds (*Turdus merula*) showed the highest mortality owing to USUV infection [18,19,25,26,27,28]. Table 1 lists the diseased and non-diseased avian species with documented USUV infections along with their native geographic locations. USUV infection in the listed avian species was determined by immunohistochemistry, reverse transcription-PCR, indirect immunofluorescence assay, ELISA, and plaque reduction neutralization assay [18,19,25,26,27,28].

**Table 1:** Avian species infected with USUV in Europe.

*Species*	Common Name	Country (year)	References
*Dendrocopos major*	Great spotted woodpecker	Belgium (2014)	[29]
*Pyrrhula pyrrhula*	Bullfinch		
*Columba livia domestica*	Domestic pigeon	Greece (2014)	[30]
*Turdus philomelos*	Song thrushes	Spain (2012)	[31]
*Turdus merula*	Eurasian blackbird	Italy (2010–2011)	[18,19,25,26,27,28]
		Germany (2011)	
		Hungary (2003–2006)	
		Austria (2001–2005)	
*Alcedo atthis*	Common kingfisher	Germany (2011)	[26]
*Serinus canaria domestica*	Canary		
*Alectoris rufa*	Partridge	Italy (2010–2011)	[18,19]
*Asio otus*	Long-eared owl		
*Caprimulgus europaeus*	Nightjar		
*Garrulus glandarius*	Eurasian jay		
*Larus michahellis*	Yellow-legged gull		
*Pica pica*	Eurasian magpie		
*Streptopelica decaocto*	Eurasian collared dove		
*Ardea cinerea*	Grey heron	Germany (2011)	[18,19,26]
*Merops apiaster*	Eurasian bee-eater	Italy (2010–2011)	
*Passer domesticus*	House sparrow		
*Picus viridis*	Eurasian green woodpecker		
*Sturnus vulgaris*	Common starling		
*Strix nebulosa*	Great grey owl	Germany (2011)	[26,27]
		Austria (2001–2002)	
*Gallus gallus domesticus*	Chicken	Italy (2007–2009)	[14,32,33,34]
		Switzerland (2006–2007)	
		England (2006)	
*Spheniscus humboldti*	Humboldt penguin	Switzerland (2006–2007)	[34]
*Phoenicopterus ruber*	Greater flamingo		
*Dacelo novaeguineae*	Laughing kookaburra		
*Ciconia ciconia*	White stork	Austria (2006–2007)	[34]
*Leptoptilos crumeriiferus*	Marabou stork		
*Neophron percnopterus*	Egyptian vulture		
*Bubo bubo*	Eurasian eagle owl		
*Bubo scandiacus*	Snowy owl		
*Strix uralensis*	Ural owl		

## 3. USUV Surveillance from Africa to Europe

Following its identification in South Africa in 1959, USUV was reported in other African countries, including Central African Republic in 1969 (CAR-1969) and 1981 (CAR-1981), in Senegal in 1974 (Kedougou-1974), 1993 (Barkedji-1993), and 2007 (Barkedji-2007) [6,22,35,36], and in Tunisia in 2014 [37]. Since its introduction to Africa, it had typically been isolated from mosquitoes and had never been associated with serious illness in mammals. It had been isolated from mammals two times: the first instance was in an African furred rat (*Praomys* sp.), and the second instance was in a man (CAR-1981) who exhibited fever and rash [6]. It is not known whether USUV originated in Africa or was introduced into this continent. Therefore, it is essential to gain a better understanding of the geographical distribution, ecology, epidemiology, and genetic diversity of this virus in Africa.

In 2001, the emergence of USUV was confirmed in Europe after a considerable die-off of Eurasian blackbirds (*T. merula*) in Vienna, Austria [7]. In subsequent years, USUV was found in several other European countries, including Hungary (2003–2006) [25], Switzerland (2006) [11], Spain (2006–2009) [10,17], Italy (2009) [16,38], Germany (2013) [15], and Belgium (2014) [29], with virus isolation from mosquitoes, birds, and bats.

**Figure 1:**
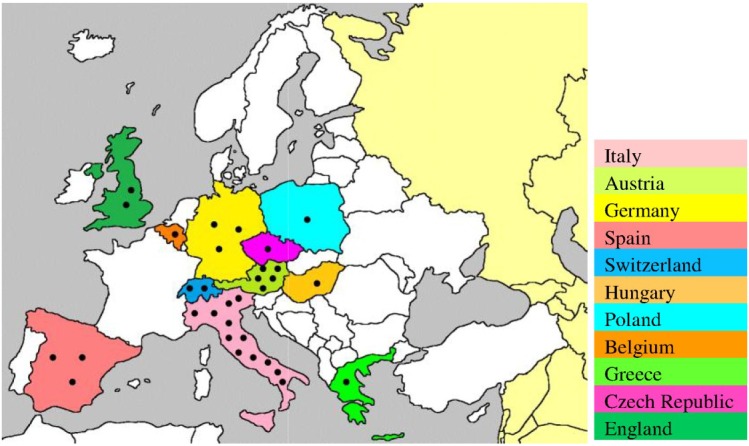
Geographic locations of USUV-related epidemiological studies on birds and mosquitoes in Europe. These studies were conducted using immunohistochemistry, reverse transcription-PCR, indirect immunofluorescence assay, ELISA, and plaque reduction neutralization assay [10,11,25,29,30,31,32,33,38,39,40,41,42,43,44]. Each epidemiological study is indicated by ●.

Moreover, USUV infection has also been demonstrated serologically in birds in England (2001–2004) [32], Czech Republic (2005) [39], Spain (2003–2006) [40], Poland (2006) [41], Switzerland (2006) [33], Germany (2007) [42], Italy (2007) [43], and Greece (2014) [30]. The recurrence of the virus in Italy (2010–2011) [18,19], Germany (2011) [26], Spain (2012) [31], and Czech Republic (2011–2012) [44] suggests persistence of the transmission cycle in the affected areas, possibly through overwintering mosquitoes [45]. In addition to wild birds, the prevalence of USUV has also been reported in birds of the zoological parks of Austria and Switzerland [34]. The broad immunological cross-reactivity between USUV and other flaviviruses could hamper the interpretation of results from serological-based studies on birds. Therefore, there is need for the development of standardized laboratory tests using validated methods that enable the differentiation of infections caused by USUV from those caused by antigenically related flaviviruses. The locations of the epidemiological studies confirming the presence of USUV in Europe are shown in Figure 1.

Interestingly, similar blackbird deaths had also been reported in Italy in 1996 [8], as were in Austria in 2001 [7]. However, the virus responsible for those deaths was unknown at that time. Because this blackbird die-off event was only reported in a local Italian veterinary journal [8], it failed to get worldwide attention. In 2013, the partial nucleotide sequence of that unknown virus was compared with the Austrian strain (Vienna-2001), revealing complete sequence identity [9]. Based on these data, USUV emergence in Europe was much earlier than previously thought.

## 4. USUV Infection in Humans

In addition to avian species, USUV has also been detected in humans. As previously discussed, the first case affecting a human was reported in Central African Republic in 1981 in a man with fever and rash [6]. Later, this strain was sequenced completely and designated CAR-1981 [46]. However, the history of the first human case of USUV-related illness is not very old in Europe. In 2009, the first European case was reported in Italy in a woman suffering from meningoencephalitis [12]. The analysis of cerebrospinal fluid by heminested RT-PCR assay that targets the prM and NS5 genes of flaviviruses, proved it a flavivirus infection. The nucleotide sequence of the yielded amplicon showed 98% identity to both Austrian (Vienna-2001) and Hungarian (Budapest-2005) strains and, thus, revealed the first human case of USUV-related neuroinvasive illness in Europe [12]. In addition, in 2009, USUV was isolated in Vero E6 cells from the blood of another Italian female patient who had undergone orthotropic liver transplantation [13]. This USUV-related viremia was confirmed by heminested RT-PCR assay targeting the NS5 gene of flaviviruses and subsequent identification by sequencing, which showed 98% identity to the USUV genome sequences available in GenBank [13]. This strain was sequenced completely and designated Bologna-2009 [47].

In 2012, a serological surveillance program was conducted in humans in southwest Germany [48]. A total of 4200 human serum samples were analyzed by an immunofluorescence assay for the detection of antibodies reacting with USUV antigen. All samples that were positive by this assay were double-checked by a commercially available USUV-specific IgG-capture ELISA kit. Among them, only one sample showed neutralizing antibodies against USUV, indicating a low and asymptomatic prevalence of USUV in Germany. A similar asymptomatic prevalence was reported in Italy, where four of 359 healthy blood donors tested positive for USUV-specific antibodies [49]. In 2013, neutralizing antibodies against USUV have also been detected in three patients in Croatia, suspected with WNV-related neuroinvasive infection [50].

Further studies should be implemented to assess the real risk of USUV infection in humans and to establish the usefulness of bird surveillance as a predictive marker for a USUV outbreak in humans.

## 5. Cellular Tropism and Pathogenesis of USUV

USUV can infect cells of various tissues types derived from humans and a wide variety of animal species [51]. Bakonyi *et al.* investigated the susceptibility of various cell lines and cultures to USUV infection that include HeLa (human), Vero (simian), ED (equine), PK-15 (porcine), RK-13 (lapin), MDBK (bovine), MDCK (canine), DK (canine), CR (feline), BHK-21 (hamster), BF (hamster), C6 (rat), TH1 (turtle), primary goose embryo fibroblasts, and horse kidney cells [51]. Among them, Vero, PK-15, and goose embryo fibroblast cells developed cytopathic effects, indicating the suitability of these cells for diagnostic purposes. However, viral multiplication was detected in all mammalian cells by immunohistochemistry [51]. This difference in pathogenesis might have been influenced by several factors, including the role of defective interfering particles, immune response, and host resistance genes [52]. USUV has also been detected in brain, heart, liver, kidney, lungs, and intestinal tissues of laboratory infected mice [53] and natural infected birds [54], and this tissue tropism is similar to WNV [55]. However, demyelination of infected neurons was found to be a unique feature of USUV infection [53]. Further investigations involving different mammalian species will be important to estimate possible threat to domesticated animals and human population.

Autophagy is an important cellular pathway that contributes important roles in viral infections and pathogenesis [56]. In relation to genus *Flavivirus*, autophagy has been associated with multiple aspects of replication and pathogenicity of some members of this genus, including DENV [57], JEV [58], and USUV [59]. Some viruses, including USUV, can take advantage of autophagic process by incorporating the components from this cellular pathway in their own replication [59,60]. Upon USUV infection, the unfolded protein response due to Xbp-1 mRNA splicing and cytoplasmic aggregation of lipidated form of microtubule-associated protein 1 light chain 3 have been observed [59], which are considered as markers of autophagosome formation during viral infections [61]. Treatment with rapamycin, an inductor of autophagy, resulted in an increase in viral titer, whereas modulation of this pathway with inhibitors, wortmannin or 3-methyladenine resulted in a decrease in viral titer [59]. These findings provide the basis for the design of new antiviral therapies against USUV.

## 6. Genomic Structure and Phylogenetic Analysis of USUV

**Figure 2:**
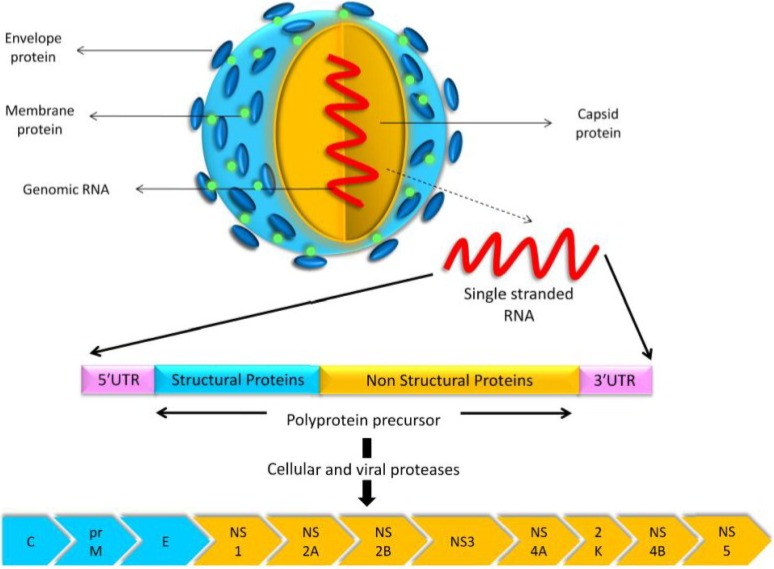
USUV model, its gene structure, and the proteins encoded by its genome. The polyprotein precursor is cleaved by cellular and viral proteases to yield three structural proteins (C, prM, and E) and eight non-structural proteins (NS1, NS2A, NS2B, NS3, NS4A, 2K, NS4B, and NS5).

USUV is a small, spherical, enveloped virus with a diameter of 40–60 nm [62]. It has an 11-kb positive-sense, single-stranded RNA genome with a 5′ cap, but without a 3′ poly-A tail [62]. Similar to other flaviviruses, the genome contains a unique open reading frame spanning nucleotides 97–10,401. The USUV open reading frame encodes a polyprotein precursor of 3434 amino acid residues, which undergoes proteolytic cleavage by both viral and cellular proteases to yield three structural and eight non-structural (NS) proteins (Figure 2). The structural proteins—core (C), pre-membrane (prM), and envelope (E)—contribute to the viral structural elements, whereas the NS proteins NS1, NS2A, NS2B, NS3, NS4A, 2K, NS4B, and NS5, regulate viral replication [62].

Phylogenetic analyses have been used to explore the genetic relationship among flaviviruses. In 2004, a phylogeny of the genus *Flavivirus* was established including one African (SouthAfrica-1959) and one European (Vienna-2001) strain of USUV [62]. This study was carried out by conducting both complete genome as well as polyprotein precursor sequence analyses [62] rather than partial nucleotide or amino acid sequence analyses, as was done previously [2,63,64]. These two USUV strains are 97% and 99% identical at nucleotide and amino acid levels, respectively [62]. When comparing USUV to other JEV serocomplex viruses, the closest relative is MVEV that exhibits 73% and 82% identity at the nucleotide and amino acid levels, respectively. JEV and WNV exhibit 71% and 68% identity with USUV at the nucleotide level and 81% and 75% at amino acid level, respectively [62].

**Figure 3:**
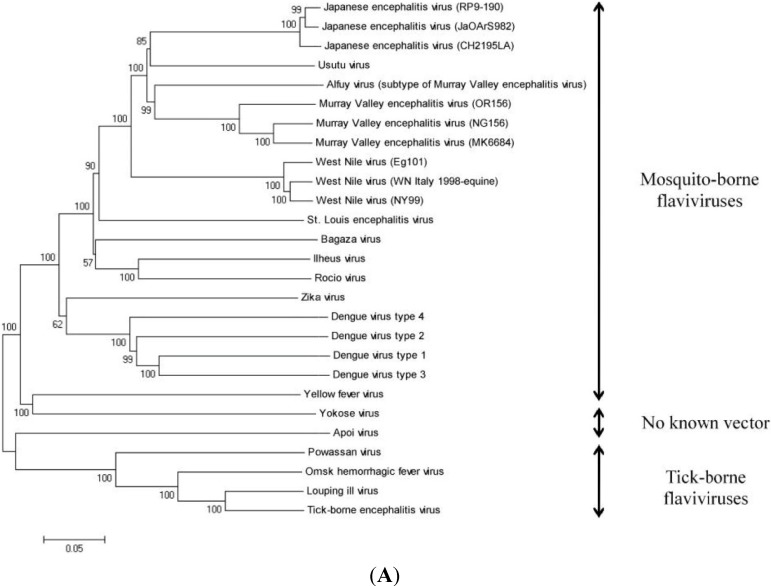

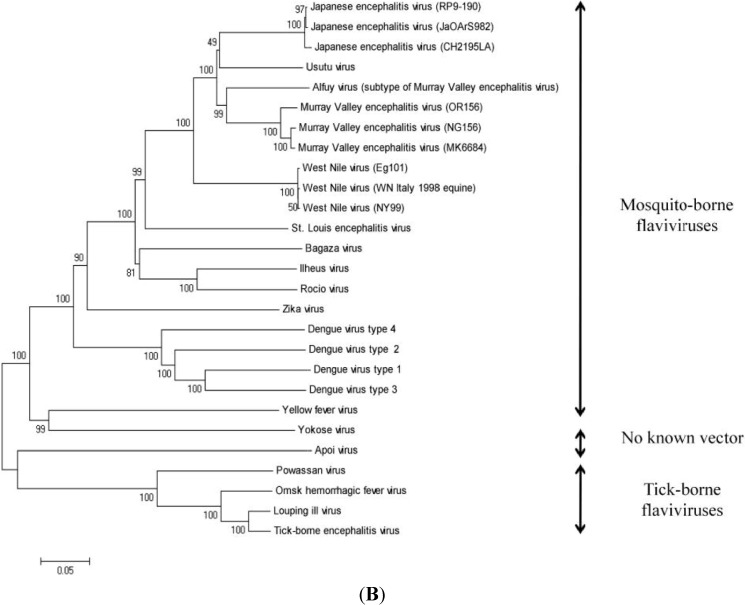
(**A**) Phylogenetic tree based on complete genome sequence analysis. This figure illustrates the close genetic relationship between USUV and MVEV, WNV, and JEV. The relative accession numbers of selected flavivirus sequences are listed in Table 2. The number at each node is the percentage of 1000 bootstrap replicates. (**B**) Phylogenetic tree based on polyprotein precursor sequence analysis. This figure illustrates the close genetic relationship between USUV and MVEV, WNV, and JEV. The relative accession numbers of selected flavivirus sequences are listed in Table 2. The number at each node is the percentage of 1000 bootstrap replicates.

The phylogeny of important flaviviruses, including USUV, based on complete genome and polyprotein precursor sequence analyses is shown in Figure 3. The phylogenetic trees are constructed by the neighbor-joining method using MEGA [65]. The relative accession numbers of selected sequences are listed in Table 2.

**Table 2:** Complete genome sequences and complete polyprotein precursor sequences used for the phylogenetic analyses.

Virus Name	Nucleotide Accession Number	Protein Accession Number
Alfuy virus	AY898809	AAX82481
Apoi virus	NC_003676	NP_620045
Bagaza virus	HQ644144	AEI27245
Dengue virus type 1	AF309641	AAK62993
Dengue virus type 2	M29095	AAA42941
Dengue virus type 3	AY679147	AAT79552
Dengue virus type 4	AF326573	AAK01233
Ilheus virus	KC481679	AGJ84083
Japanese encephalitis virus (CH2195LA)	AF221499	AAF34186
Japanese encephalitis virus (JaOArS982)	M18370	AAA81554
Japanese encephalitis virus (RP9-190)	KF907505	AHK05344
Louping ill virus	KF056331	AGN32859
Murray Valley encephalitis virus (MK6684)	KF751869	AIA58169
Murray Valley encephalitis virus (NG156)	KF751870	AIA58170
Murray Valley encephalitis virus (OR156)	KF751871	AIA58171
Omsk hemorrhagic fever virus	AY193805	AAP29989
Powassan virus	L06436	AAA02739
Rocio virus	AY632542	AAV34158
St. Louis encephalitis virus	NC_007580	YP_001008348
Tick-borne encephalitis virus	KF151173	AGP05331
Usutu virus	AY453412	AAS59401
West Nile virus (Eg101)	AF260968	AAG02039
West Nile virus (NY99)	DQ211652	ABA62343
West Nile virus (WN Italy 1998-equine)	AF404757	AAM81753
Yellow fever virus	DQ235229	ABB69689
Yokose virus	NC_005039	NP_872627
Zika virus	AY632535	AAV34151

## 7. Genetic Diversity among Different USUV Strains

Many USUV strains have been identified and sequenced. Of these strains, the genomes of only 15 have been sequenced completely (Table 3), whereas the remaining strains have been only partially sequenced [15,46,47,66]. When compared with the reference strain (SouthAfrica-1959), all of the completely sequenced strains, except CAR-1969, exhibit 97% and 99% similarity at the nucleotide and amino acid levels, respectively, whereas CAR-1969 shares 81% nucleotide and 95% amino acid similarity. These data were confirmed by BLAST analysis (http://www.ncbi.nlm.nih.gov/blast).

**Figure 4:**
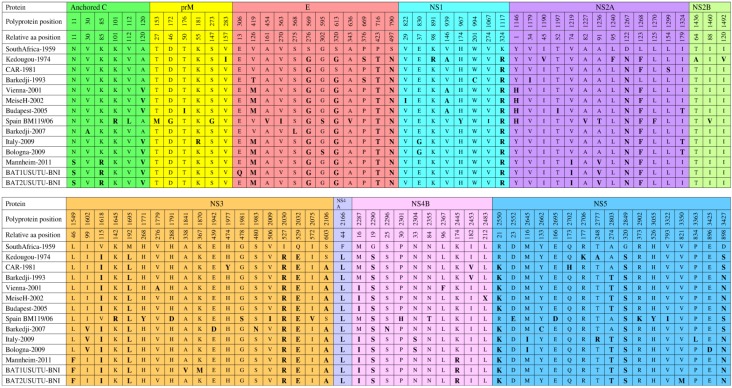
Comparison of polyprotein precursors of fully sequenced USUV strains. Anchored C protein (1–126), prM protein (127–293), E protein (294–793), NS1 protein (794–1145), NS2A protein (1146–1372), NS2B protein (1373–1503), NS3 protein (1504–2122), NS4A protein (2123–2248), 2K protein (2249–2271), NS4B protein (2272—2529), and NS5 protein (2530–3434). All amino acid substitutions are shown with bolded and enlarged letters.

The comparative analysis of the polyprotein precursor of all completely sequenced strains, except CAR-1969, to the reference strain revealed amino acid substitutions at specific positions (Figure 4) [15,26,46,47]. A total of 11 amino acid substitutions (G569S, T716P, N790S, R1117K, N1267D, L1695M, R2030I, E2032Q, L2166F, S2290G, and S2849G) are common in all strains. However, certain substitutions are seen in particular strains, which may contribute to yet unknown strain-specific characteristics. These distinct substitutions are present in Kedougou-1974 (I283V, R891K, V1190I, F1240L, A1436T, V1492I, and K2706R), CAR-1981 (S1299L, Y1977H, and H2702Q), Barkedji-1993 (C994W and I1179V), Vienna-2001 (A1779V and F2367L), MeiseH-2002 (I822V and X2483L), Budapest-2005 (I176T and I1197T), Spain BM119/06 (R101K, L112V, M153T, G172D, G273S, V454A, I563V, V636A, Y967H, I1067V, V1227A, F1270L, V1460I, R1645K, Y1771H, D1791H, S1981G, I2009V, V2075I, H2301P, T2355N, E2552D, D2695E, K2902R, Y3055H, and I3322V), Barkedji-2007 (A30V, L568S, D1942E, N1983S, N2296S, and C2662Y), Italy-2009 (R181K and L3363P), Bologna-2009 (D3425E), BAT1USUTU-BNI (Q306E, V1841A, and M1870K), and BAT2USUTU-BNI (M3350V). Interestingly, S595G and D3425E substitutions in Bologna-2009 are considered important because they might have played a role in promoting the human-specific neuroinvasive capacity of this virus [47]. These substitutions are not seen in CAR-1981, which was associated with symptoms, such as fever and rash in a man [6]. Therefore, when comparing the two strains affecting humans, the two Bologna-2009 substitutions (S595G and D3425E) may contribute to the difference in virulence between them. Moreover, the Bologna-2009-related substitutions (S595G and D3425E) are common to some other flaviviruses (DENV, JEV, WNV, and MVEV) that also threaten human health [47]. DENV-2 isolates in Southern-Asia associated with human encephalitis [67] and Bologna-2009 have the same amino acid (serine) at position 595 in the DIII-Ir domain of the E protein [47]. Domain DIII of the E protein of flaviviruses is the likely receptor binding domain and the major determinant of virus cellular tropism [68]. Specific amino acid substitutions within Domain DIII of WNV have been implicated as mediators of virus infectivity, antigenicity, and virulence [69]. In addition, the D3425E substitution in Bologna-2009 is similar to that found in certain strains of JEV, WNV, and MVEV [47]. Studies on WNV have shown that substitutions in virtually equivalent positions were associated with variation in the ability of WNV to invade the central nervous system of laboratory-infected mice [70]. Furthermore, A1236V and L1549F substitutions in Mannheim-2011, BAT1USUTU-BNI, and BAT2USUTU-BNI are also considered important because similar mutations in the related WNV modulated the host antiviral response by inhibition of interferon signaling [71].

**Table 3:** Nucleotide and amino acid sequence similarity in fully sequenced USUV strains.

Strain	Geographical	Genome	Total	Nucleotide	Amino Acid	Nucleotide	Protein
	Origin	Length (bp)	Amino Acids	Similarity %	Similarity %	Access. No	Access. No
South Africa-1959	South Africa	11064	3434	-	-	AY453412	AAS59401
CAR-1969	CAR	10745	3434	81	95	KC754958	AGP50649
Kedougou-1974	Senegal	10837	3434	97	99	KC754954	AGP50645
CAR-1981	CAR	10800	3434	97	99	KC754955	AGP50646
Barkedji-1993	Senegal	10837	3434	97	99	KC754956	AGP50647
Vienna-2001	Austria	11066	3434	97	99	AY453411	AAS59402
MeiseH-2002	Austria	11047	3434	97	99	JQ219843	AFE85504
Budapest-2005	Hungary	11065	3434	97	99	EF206350	ABP88817
Spain BM119/06	Spain	11064	3434	97	99	KF573410	AHA57377
Barkedji-2007	Senegal	10825	3434	97	99	KC754957	AGP50648
Italy-2009	Italy	11065	3434	97	99	JF266698	AEK21245
Bologna-2009	Italy	11065	3434	97	99	HM569263	AEF13245
Mannheim-2011	Germany	11003	3434	97	99	HE599647	CCD57503
BAT1USUTU-BNI	Germany	11065	3434	97	99	KJ859682	AIN76231
BAT2USUTU-BNI	Germany	11065	3434	97	99	KJ859683	AIN76232

To gain a better knowledge about these strain-specific amino acid substitutions, comparative pathogenesis studies on different USUV strains will be important using animal models.

**Figure 5:**
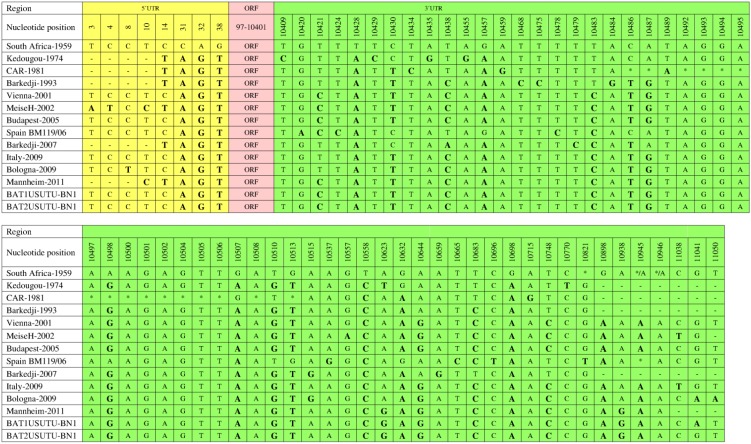
Comparison of 5ʹ UTRs and 3ʹ UTRs of fully sequenced USUV strains. 5ʹ UTR (1–96), open reading frame (ORF;97–10,401), 3ʹ UTR (10,402–11,064). All nucleotide substitutions are shown with bolded and enlarged letters. Nucleotide deletions are indicated by an asterisk (*).

The 5ʹ and 3ʹ UTRs play important roles in flavivirus genome replication and translation [72,73,74]. The first 96 and the last 666 nucleotides in the genomes of different USUV strains contribute to the 5ʹ and 3ʹ UTRs, respectively [63]. Comparative analysis of all completely sequenced USUV strains, except CAR-1969, using the reference strain as a baseline revealed various nucleotide substitutions in the 5ʹ and 3ʹ UTRs of the strains (Figure 5) [15,26,46,47]. In the 5ʹ UTRs, all strains showed common nucleotide substitutions at positions 31, 32, and 38. At position 14, only six strains (Kedougou-1974, CAR-1981, Barkedji-1993, MeiseH-2002, Barkedji-2007, and Mannheim-2011) showed a common nucleotide substitution. At position 10, MeiseH-2002 and Mannheim-2011 showed a common nucleotide substitution. However, MeiseH-2002 showed distinct nucleotide substitutions at positions 3 and 4.

In the 3ʹ UTRs, the nucleotide substitution scenario may be more revealing than in the 5ʹ UTRs. The most common nucleotide substitutions were observed at positions 10428, 10558, and 10698. At positions 10898 and 10945, only European strains showed common substitutions. The pattern of distinct substitutions also varied among different USUV strains. These distinct substitutions were observed in Kedougou-1974 (positions 10409, 10429, 10435, 10455, and 10770), Barkedji-1993 (10468, 10475, and 10484), MeiseH-2002 (10557), Spain BM119/06 (10420, 10424, 10478, 10537, 10665, and 10696), Barkedji-2007 (10479 and 10659), and Bologna-2009 (11050). CAR-1981 exhibited the most distinct features in its 3ʹ UTR with four unique substitutions (positions 10434, 10459, 10489, and 10715) and 16 nucleotide deletions (Figure 5). These deletions were not observed in the Bologna-2009 strain, which might have played a basic role in the capability of this isolate to infect and provoke disease in a human [46].

CAR-1969 is the most divergent strain of USUV, as mentioned earlier. Because it has the most diversity when comparing the USUV strains, historically it has been difficult to determine whether it should be classified as a separate viral species. The genetic distance between all USUV strains, including CAR-1969 (0.00–0.19 substitutions per site), which do not exceed those observed for other closely related viruses of the JEV serocomplex, namely WNV (0.00–0.22 substitutions per site) or JEV (0.01–0.21 substitutions per site), categorized it as an USUV strain [46]. Furthermore, cross-reactivity between SouthAfrica-1959 and CAR-1969 has also been demonstrated using complement fixation assay [75].

## 8. Diagnosis

The occurrence of human cases of USUV infection has underscored the need to develop reliable and confirmatory diagnostic tools for detection. In this regard, Cavrini *et al.* developed the first rapid, USUV-specific real-time RT-PCR assay based on Austrian and Hungarian strain sequences [76]. This assay could detect USUV in human blood and cerebrospinal fluid samples with high specificity and sensitivity, but its detection reliability was limited to a few European strains. This limitation was overcome by another recently developed real-time RT-PCR assay based on African and European strain sequences [77]. These diagnostic assays allow the detection of USUV in blood and cerebrospinal fluid samples at the viremic stage; therefore, serological testing is important to identify the infection following the viremic stage.

In 2012, Gaibani *et al.* developed the first USUV-specific IgG-capture ELISA assay for serological diagnosis of USUV [49]. This assay can detect USUV-specific IgGs in both Italian and German healthy blood donors, as mentioned earlier [48,49]; cross-reactivity between USUV and WNV was resolved by adapting a diagnostic algorithm [78] and the plaque reduction neutralization test [49]. The latter test is a current standard to discriminate between closely related flaviviruses [49]. Interestingly, in flaviviruses, cross-reactivity is higher for IgG than for IgM [79]; therefore, USUV-specific IgM-based assays must be developed. Considering that USUV infections have been documented throughout Europe and that few approaches are available for reliable detection and diagnosis, the scope of USUV-related diagnosis is wide open, and additional efforts are needed to adequately prepare for a possible large-scale USUV outbreak.

## 9. Conclusions

In Africa, USUV is typically isolated from mosquitoes and generally infects only four avian species, namely *Bycanistes sharpei* (piping hornbill), *Andropadus virens* (little greenbul), *Turdus libonyanus* (Kurrichane thrush), and *T. merula* (blackbird) [35]. However, the non-migratory behavior of these species suggests that they have not been involved in dispersing USUV throughout Europe. In Austria (2005), a serological study demonstrated the presence of USUV-specific antibodies in several migratory bird species, including *Sylvia communis* (whitethroat), *Sylvia curruca* (lesser whitethroat), *Sylvia borin* (garden warbler), *Falco tinnunculus* (kestrel), *Circus aeruginosus* (marsh harrier), *Delichon urbica* (house martin), *Acrocephalus scirpaceus* (reed warbler), *Ficedula hypoleuca* (pied flycatcher), and *Hirundo rustica* (barn-swallow) [80]. Based on their migratory habits and the presence of USUV-specific antibodies, these bird species may have played a role in the introduction of USUV into Europe, but further investigation into this possibility is still needed. Moreover, *Cx. pipiens* mosquitoes and Eurasian blackbirds (*T. merula*) are two important causes of USUV dissemination in Europe [16,17,18,19,20,21,25,26,27,28].

The migration pattern of blackbirds brings them in Eastern Asian countries, including Eastern Russia, Eastern China, Taiwan, Korea, and Japan [81], whereas *Cx. pipiens* mosquitoes are also common in Asian countries [82,83,84]. Considering these factors, USUV might be a potential threat to populations in Asia. However, competence studies of the local population of the potential vectors should be evaluated for real risk assessment in Asian countries. To prevent the emergence of USUV on a larger scale, veterinary-, human-, and entomology-based surveillance programs should be established throughout Europe.

Newly developed USUV-specific real-time RT-PCR assays and ELISA are very helpful for screening and diagnostics [49,77]. However, attaining a USUV-specific serological diagnosis will be quite challenging in certain areas where other flaviviruses occur along with USUV. Such potential cross-reactivities must be ruled out by plaque reduction neutralization test. An inactivated Japanese encephalitis vaccine formulated with Advax adjuvant has induced a cross-protective immune response against MVEV and WNV [85,86]. Because USUV is a member of the JEV serocomplex, Japanese encephalitis-Advax vaccine might be an alternate approach to control USUV infection, and, thus, threat to Asia can be reduced in JEV-immune populations. Furthermore, a recently developed WNV recombinant subviral particle vaccine showed a cross-reactive humoral response against USUV in mice [87]. A multidimensional approach is also necessary for effective USUV-related risk assessment and to determine the involvement of various elements (bird, mosquito, human, rodents, and horses) in the USUV infection cycle.
